# Laparoscopic treatment of obstructed internal supravesical hernia: A cases series and rewiev of the literature

**DOI:** 10.1016/j.amsu.2018.10.021

**Published:** 2018-10-23

**Authors:** Clementi Marco, Bonanni Luigi, Sista Federico, Vicentini Vincenzo, Salvatorelli Andrea, Di Furia Marino, Puccica Ilaria, Guadagni Stefano

**Affiliations:** a Department of Medicine, Health and Life, University of L'Aquila, Piazza S. Tommasi, 67100, L'Aquila, AQ, Italy; b Hepatobiliary Surgical Unit, San Salvatore Hospital, 67100, L'Aquila, AQ, Italy; c Department of Applied Clinical Science and Biotechnology, University of L'Aquila, Via Vetoio, 67100, L'Aquila, AQ, Italy

**Keywords:** Internal supravesical hernia, Small bowel obstruction, CT-Scan, Laparoscopy

## Abstract

**Introduction:**

internal supravesical hernia (ISH) is an exceptional subtype of internal hernia often presenting with small bowel obstruction (SBO). Its rarity makes preoperative suspicion and diagnosis very difficult in an emergency setting.

**Methods:**

we retrospectively analyzed the database of patients admitted in a single center emergency unit for small bowel occlusion (SBO) in virgin abdomen and treated by surgery from August 2013 to October 2018. The patients with intraoperative diagnosis of ISH were included in this study.

**Results:**

from 29 patients with virgin abdomen submitted to surgery for SBO, two cases of ISH were recorded. In both cases preoperative diagnosis was made by CT scan and urgent treatment was successfully performed by laparoscopy, reducing the entrapped small bowel and closing the hernia's ring by continuous suture. No intestinal resection was needed.

**Discussion:**

urgent laparotomic repair of obstructive ISH is the standard treatment although laparoscopic approach has also been described in a small number of cases. We reported our experience on two cases in which totally laparoscopic treatment was successfully performed in patients with stable hemodynamic parameters thanks to early diagnosis and limited intestinal distension. By an extensive analysis of the international literature, clinical, diagnostic and therapeutic aspects of this form of internal hernia were discussed.

**Conclusion:**

CT scan facilitates early ISH preoperative diagnosis, reducing the risk of small bowel resection and increasing the chances of minimally invasive laparoscopic treatment.

## Highlights

• Internal supravesical hernia is a rare condition presenting with acute small bowel obstruction.
• We present two cases in which laparoscopic treatment has been successfully performed.
• A complete review of the literature was also reported.

## 1 Introduction

Supravesical hernia (SH) occur in a triangular space bounded laterally by the median umbilical ligament, medially by the urachus and inferiorly by the Cooper's ligament [1]. According to the hernia's sac development, SH is classified as external or internal [2]. The most common external type protrudes through the anterior abdominal wall expanding into the inguinal canal and miming a direct inguinal hernia. On the contrary, the internal type remains within the abdomen passing into the space around the bladder: this type is extremely rare, generally described in single case-report [[3], [4], [5], [6], [7], [8], [9], [10]]. The majority of patients with internal supravesical hernia (ISH) present acute obstructive symptoms caused by small bowel strangulation. Laparotomic repair with bowel resection, has been described in several different cases [[3], [4], [5], [6], [7], [8], [9], [10], [11], [12], [13], [14], [15], [16], [17], [18], [19], [20], [21], [22]]; more rarely, laparoscopic treatment has been reported [[23], [24], [25], [26], [27]].

Patients with small bowel obstruction (SBO) and surgically virgin abdomen, have been retrospectively evaluated, detecting two cases of obstructive ISH treated by laparoscopy. The available international literature of the last years was also collected to highlight and discuss clinical, diagnostic and therapeutic aspect of this rare form of internal hernia. The work has been reported in line with PROCESS criteria [28].

## 2 Methods

The database of patients submitted to surgery for SBO in the Surgical Units of University of L'Aquila from August 2013 to October 2018, has been retrospectively analyzed. The patients with intraoperative diagnosis of ISH were included in this study. Using PubMed and Goggle Scholar database, we reviewed the international literature using the MeSH terms “paravesical”, “supravesical”, “internal hernia”, “small bowel occlusion” to identify the cases treated by surgery after 1995. This study has been registered on the Research Registry with the Unique Identifying Number 4265.

## 3 Results

Twenty-nine patients with virgin abdomen underwent surgery for SBO during the analyzed period. Two cases of ISH were recorded. Both procedures were performed by two surgeons skilled in mini-invasive management of abdominal urgencies (MC, LB). From the available international literature, we identified 26 previous cases of internal supravesical or paravesical hernia treated by urgent laparotomic or laparoscopic surgery (Table 1).

**Table 1:** Literature review from 1995.

Author/Reference	Age/Sex	Presenting complaint	Radiological study	Pre-op diagnosis	Operative methods	Intestinal resection	Resection of the hernia sac	Treatment of the hernia rings	Follow-up (months)
Korsoy F^3^	78/M	I.O.	RX	SBO	Laparotomy	No	No	Closure	20
Ohe S^4^	73/M	I.O.	None	SBO	Laparotomy	Yes	No	Closure	#
Tanaka S^5^	70/M	I.O.	TC	Internal hernia	Laparotomy	Yes	Yes	Closure	#
Sasaya T^6^	74/M	I.O.	Rx/TC	SBO	Laparotomy	No	No	Closure	#
Gorgun E^23^	#	I.O.	TC	Small bowel invagination	Laparoscopy	No	No	Closure	#
Watanabe T^8^	78/M	I.O.	TC	Inguinal hernia	Laparotomy	Yes	No	Closure	#
Mehran A^24^	53/M	I.O.	Rx/TC	Internal hernia	Laparoscopy	Yes	No	Closure	#
Sozen I^7^	43/M	I.O.	None	SBO	Laparotomy	No	Yes	None	#
Sato T^9^	86/F	I.O.	TC	Obturator hernia	Laparotomy	Yes	No	Closure	#
Jan YT^10^	75/M	I.O.	Rx/TC	Internal hernia	Laparotomy	No	No	Closure	#
BouassidaM^13^	58/M	I.O.	TC	IH	Laparotomy	Yes	No	Closure	#
	36/M	I.O.	RX	SBO	Laparotomy	No	No	Closure	#
Saravan B^11^	62/M	I.O.	RX	SBO	Laparotomy	Yes	No	Closure	#
Cissé M^12^	60/M	I.O.	Rx/TC	Small bowel intussusception	Laparotomy	No	No	Closure	#
Schwarz L^25^	76/M	I.O.	TC	Small bowel intussusception	Laparoscopy	Yes	Yes	Closure	6
Akaike H^14^	70/M	I.O.	TC	Internal hernia	Laparotomy	#	#	#	#
Watanabe Y^15^	60/M	I.O.	RX/TC	Internal hernia	Laparotomy	No	#	#	#
Asanuma K^26^	52/M	I.O.	TC	Small bowel tumor	Laparoscopy	No	No	Closure	#
Jerraya H^16^	56/M	I.O.	RX/TC	Internal hernia	Laparotomy	No	No	Closure	#
Adamou H^17^	49	I.O.	Rx	SBO	Laparotomy	No	Yes	Closure	#
Khalid S/2015^18^	62/M	I.O.	Rx/TC	Small bowel volvulus	Laparotomy	No	No	Closure	6
Fuentes SA^20^	74/M	I.O.	TC	SBO	Laparotomy	No	No	Closure	#
Sardiwalla II^22^	35/M	I.O.	RX	SBO	Laparotomy	No	No	Closure	#
Takahara Y^27^	48/M	I.O.	TC	Small bowel volvulus	Laparoscopy	#	#	#	#
Morimoto M^19^	74/M	I.O.	TC	Strangulated inguinal hernia	Anterior approach	Yes	#	Closure	22
Diez BL^21^	67/M	I.O.	Rx/TC	Small bowel bridle	Laparotomy	Yes	No	Closure	#

I.O.: intestinal obstruction, S.B.O.: small bowel obstruction.

### 3.1 Case 1

**Fig. 1:**
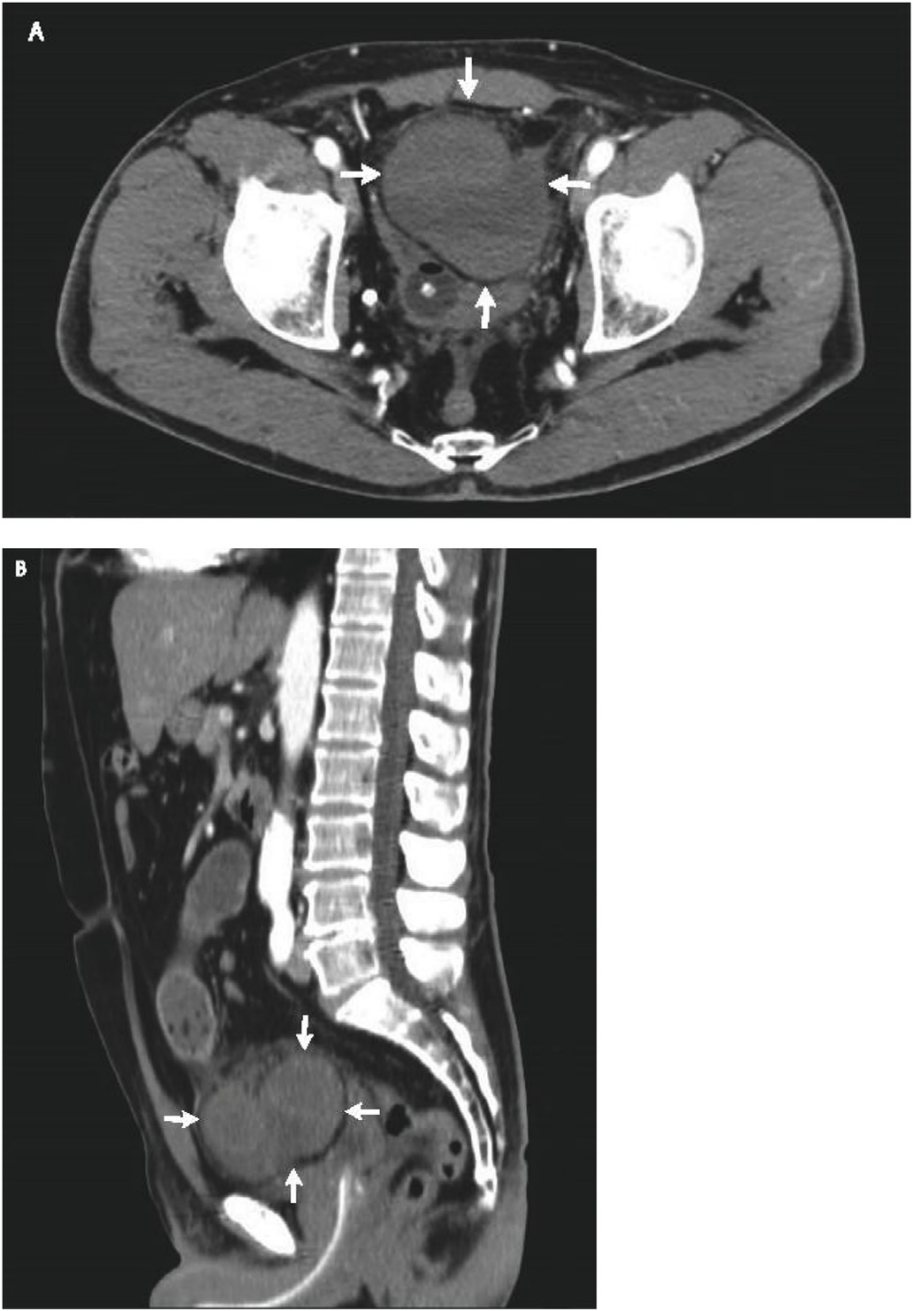
CT scan showed saclike mass of clustered dilated bowel loops (arrows) descending downward anterior and laterally to the bladder, compressing it. Sagittal (A) and coronal (B) sections.

**Fig. 2:**
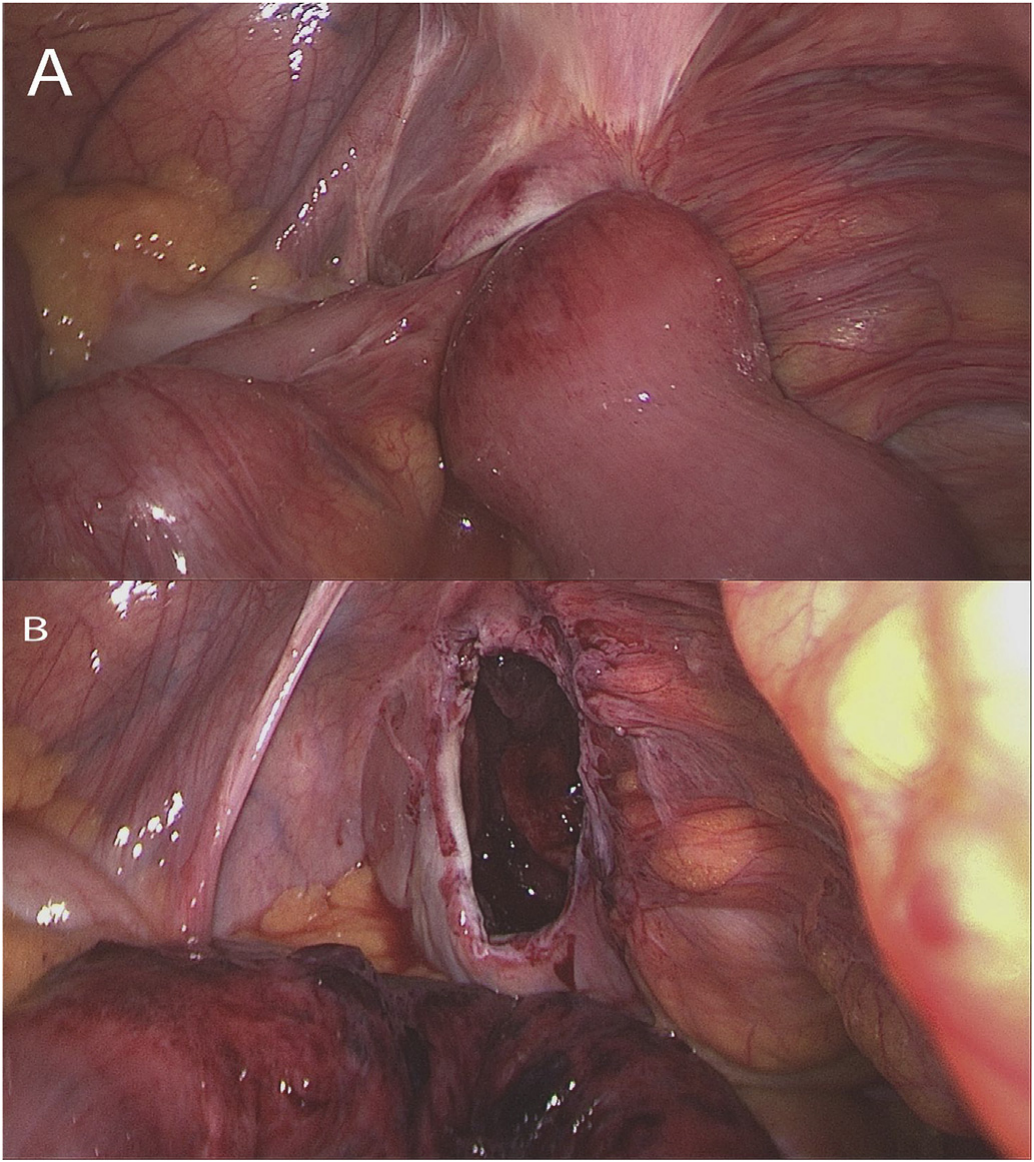
Intraoperative laparoscopic views of incarcerated small bowel (A) and hernial orifice after small bowel reduction (B).

A 48-year-old healthy man with BMI 30 was admitted to the emergency department with a 12-h duration history of abdominal pain and distension with bilious vomiting. The patient had no history of previous similar attacks. On examination, the patient was dehydrated, afebrile with a pulse minute rate of 98 and blood pressure of 130/80. His abdomen was distended, tympanic on percussion and tinkling bowel sounds were auscultated. The clinical suspect of bowel occlusion was confirmed by an abdominal X-ray in the upright position that revealed multiple fluid levels without free air. The abdominal and pelvic CT scan with intravenous contrast identified an SBO with a transizional zone in the right lower abdomen. Below the transitional zone there was a saclike mass of clustered dilated bowel loops descending downward into the prevesical space and compressing the anterolateral wall of the bladder (Fig. 1). The patient, informed about the radiological suspect of internal hernia, provided informed consent to laparoscopic approach. A laparoscopy by a three trocars technique (12 mm trocar at navel and two 5 mm trocars at bilateral abdominal flank) was performed confirming the radiological diagnosis of obstructive supravesical hernia involving the terminal ileum (Fig. 2A). With the patient in Trendelemburg's position, the entrapped small bowel was gently reduced revealing a hernia's ring of 2 cm × 4 cm with a sac running laterally and anteriorly to the bladder (Fig. 2B). The segment of incarcerated intestine was found to be viable for which bowel resection was not required. The internal surface of the sac was cauterized by bipolar device and the hernia's ring was closed with 2/0 Polydioxanone (PDS) running suture. After an uneventful recovery, the patient was discharged on the four post-operative day. Two months later, the patient presented with a bulging mass in the right inguinal area and had repair of direct inguinal hernia. After 23 months follow-up the patient did not develop clinical or radiological signs of recurrences of supravesical hernia.

### 3.2 Case 2

**Fig. 3:**
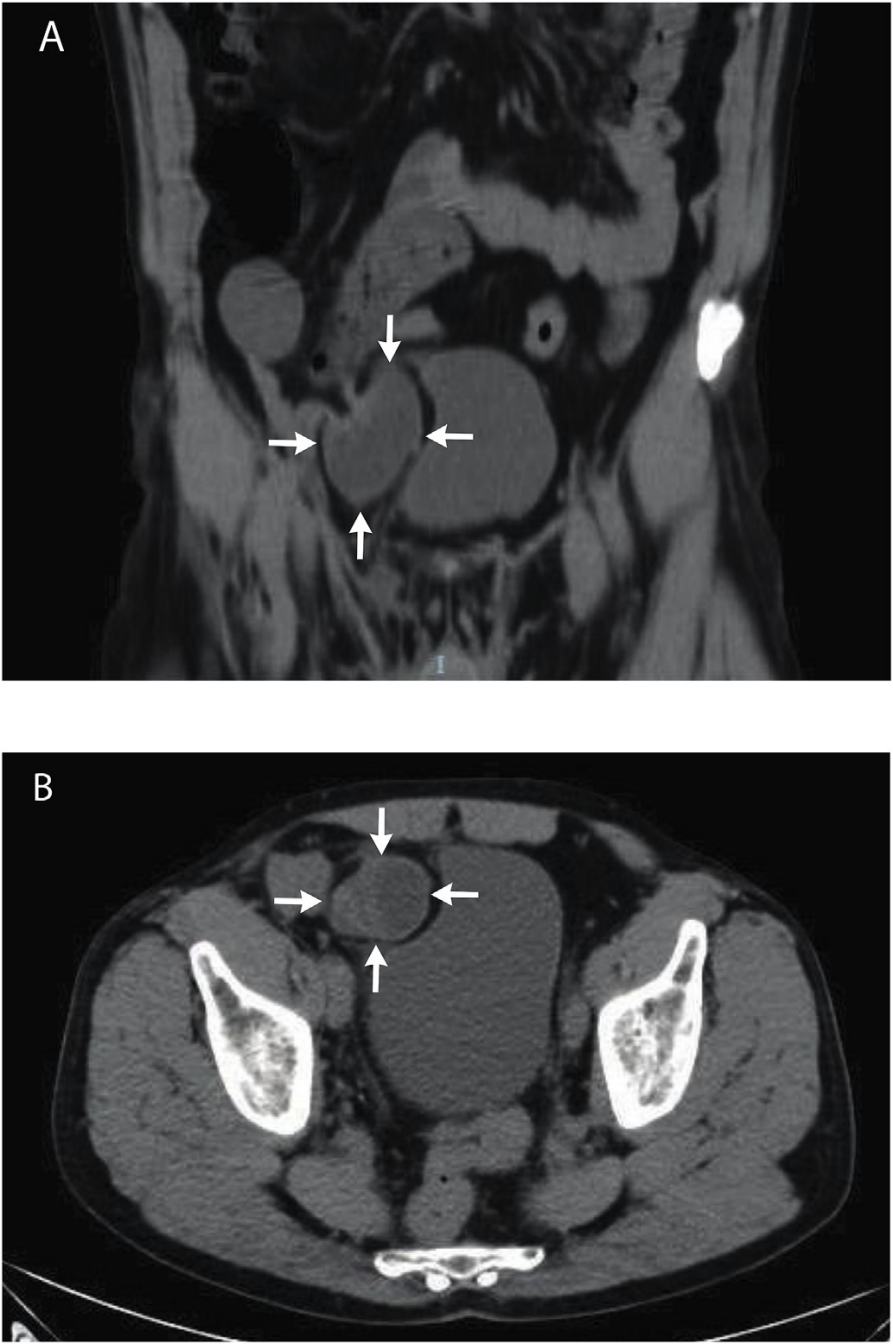
Abdominal CT scan showing a saclike mass (arrows) of bowel loops within a hernia sac below the transitional zone, indicating an internal hernia. Coronal (A) and sagittal (B) section.

A 65-year-old healthy man with BMI 32 was admitted to the emergency department. He referred 6-h before symptoms, following heavy cough, mostly related to abdominal pain and nausea, with one episode of bilious vomiting but no clinical evidence of heavy abdominal distension. The patient had no history of previous similar episodes. Clinical examination revealed dehydratation, high body temperature (38.2 °C), pulse rate of 98/minute and mild hypotension (blood pressure 110/60 mmHg). His abdomen was little distended, mostly tympanic on percussion and tinkling bowel sounds were auscultated especially on the right inferior quadrants. To confirm the suspect of bowel occlusion, an abdominal X-ray in the upright position was performed revealing multiple fluid levels without free air in the peritoneal cavity. Abdominal and pelvic CT scan without intravenous contrast identified a small bowel obstruction with a transitional zone in the right lower abdomen, starting from a saclike mass of clustered dilated bowel loops descending into the prevesical space and compressing the anterolateral wall of the bladder (Fig. 3). The patient, informed about the radiological suspect of internal hernia, provided informed consent to a minimal invasive approach. Laparoscopy revealed a not necrotic ileal incarcerated loop in a hernia's ring of 1.5 cm × 3 cm with a sac running laterally and anteriorly to the bladder. The same surgical technique of the first case was adopted. Recovery was rapid and uneventful. After 18 months follow-up, the patient did not develop clinical or radiological signs of supravesical hernia recurrence but showed a left direct inguinal hernia for which was submitted to surgery.

## 4 Discussion

Internal prevesical hernia, judging from the number of cases reported, is a rare form of internal hernia. Rising from the supravesical fossa, it may develop anteriorly or laterally to the bladder, protruding into prevesical space of Retzius or into the paravesical space, respectively. This different way of bulging generated various titles with which it has been mentioned in the literature: prevesical, supravesical, anterior retroperitoneal, properitoneal, paravesical [29]. The first internal supravesical hernia was described in 1814 by Ring [30]. Since then, few cases have been reported in literature. Our review identified 26 cases (Table 1). Unfortunately, the majority of these reports are available only like short communication or in non-English language making the data collection difficult and often incomplete. ISH generally occur in males over 50 years of age. Probably, in predisposed subjects, the bladder filling causes a failure of the integrity both of transversus abdominis aponeurosis and trasversalis fascia, generating a supravesical peritoneal diverticula [1]. Quite all symptomatic patients had digestive symptoms such as abdominal pain and vomiting caused by small bowel obstruction. Ischemic necrosis of the small bowel may occur as a consequence of strangulation. Urinary symptoms like dysuria has also been described due to bladder compression by dilated small bowel loops [31].

These data were confirmed in our patients complaining symptoms suggestive for SBO beginning 6 and 12 h respectively before the access to emergency department. Although characteristic CT findings of strangulated supravesical internal hernia have been described [6], the uncommon recognition of this hernia, make its preoperative suspicion and diagnosis quite difficult in an emergency setting. Almost in all reviewed cases, the diagnosis was made at operations despite CT scans were performed in more than 70% of patients. Only 25% of patients submitted to a preoperative CT scan, received the diagnosis of strangulated internal hernia, correctly made in both our patients. Preoperative diagnosis helps the surgeon in choosing appropriate timing and approach.

Quite almost reported cases were treated by open laparotomy [[3], [4], [5], [6], [7], [8], [9], [10], [11], [12], [13], [14], [15], [16], [17], [18],[20], [21], [22]], whereas only few cases were repaired by laparoscopic surgery [[23], [24], [25], [26], [27]] as described in our experience. According to other Authors [32,33], we believe that in case of early diagnosis and in patients with stable hemodynamic parameters, through magnification of the image, laparoscopy gives a better vision of the operating field, facilitating the surgeon's work.

Like for others rare conditions, the surgical treatment is no standardized. Reduction of the herniated loops with closure of the hernia's sac using continuous or interrupted sutures is considered sufficient whereas complete or partial resection of the sac was considered unnecessary and performed in only few of laparotomic reported cases [[20], [21], [22], [23], [24], [25], [26], [27]]. In our experience, we simply closed the edge of the ring by continuous nonabsorbable suture without removing the hernia sac. In both cases, we treated the internal surface of the sac by bipolar cauterization, trusting to the fact that bladder distention may quickly close the space let free from the retraction of the small bowel. We retain that sac dissection is dangerous with risk of bladder damage.

Even if prosthetic repair is the gold standard for inguinal and incisional hernias, mesh reinforcement was not adopted in previously reported cases, without late recurrences [[20], [21], [22], [23], [24], [25], [26], [27]], but data concerning patient's follow-up were inadequate. Our patients did not develop clinical or radiological signs of recurrences respectively 23 and 13 months after surgery.

When prompt exploration is not carried out, the strangulated part of small bowel may become gangrenous much to make bowel resection necessary. In a series of 25 patients with internal hernia submitted to surgery, delayed laparotomy time (>3 days after the onset of symptoms) have increase significantly the number of bowel resections and their related morbidity [34]. Small bowel resection, when necessary, can be done also during laparoscopy using a totally intracorporeal anastomosis or performing an external anastomosis trough accessory mini-laparotomy or port site enlargement. Both our patients received early surgical treatments (within 24 and 18 h from SBO development), avoiding bowel irreversible ischemic lesions.

Potential limitations of this study were the small number of patients and the relatively short follow-up period, in relation to the risk of ISH recurrences.

In conclusion, we believe that strangulated ISH should be considered as possible cause of an unexplained small bowel obstruction, especially in adult male without previous surgery. CT scan in emergency setting may help the surgeon in choosing approach and timing of the procedure. The surgeon with experience of laparoscopic inguinal hernia repair, should consider in patients with stable hemodynamic parameters the operative mini-invasive technique as alternative to conventional laparotomy, in consideration to the advantages in terms of postoperative pain, hospital stay, cosmetic outcomes and laparotomy related morbidity, especially in obese [35] and young patients.

## Consent

Written informed consent was obtained from the patients for publication and any accompanying images.

## Provenance and peer review

Not commissioned, externally peer reviewed.

## Ethical approval

Not applicable.

## Source of funding

All the authors declare that they have no source of funding.

## Author contribution

Clementi M and Guadagni S contributed the original idea and steasure of the manuscript.

Clementi M, Bonanni L and Di Furia M contributed by conceptualization and performing the surgical procedures.

Sista F, Salvatorelli A, Puccica I and Vicentini V contributed by collecting all the data.

Guadagni S. and Clementi M contributed by conceptualization and revision of the manuscript.

The final manuscript has been read and approved by all named authors and that there are no other persons who satisfied the criteria for authorship but are not listed. We further confirm that the order of authors listed in the manuscript has been approved by all of us.

## Conflicts of interest

All the authors declare that they have no conflict of interest.

## Trial registry number

Researchregistry4265.

## Guarantor

Prof Marco Clementi, MD.

## Consent

There is no need for ethical approval because it is a case report. Written informed consent was obtained from the patients for publication of this cases series and any accompanying images.
